# The Association Between Structural, Performance, and Community Factors and the Likelihood of Receiving a Penalty Under the Hospital Readmissions Reduction Program (Fiscal Year 2013–2019)

**DOI:** 10.1089/heq.2019.0123

**Published:** 2020-04-20

**Authors:** Jason N. Mose, Neela K. Kumar

**Affiliations:** ^1^ Department of Health Services and Information Management, East Carolina University, Greenville, North Carolina, USA.; ^2^ Department of Health Policy and Management, University of North Carolina at Chapel Hill, Chapel Hill, North Carolina, USA.

**Keywords:** Hospital Readmission Reduction Program, HRRP, HRRP hospital penalty, 30-day readmission, unplanned hospital readmissions, Medicare's readmission reduction program

## Abstract

**Purpose:** Little is known about the role of structural, performance, and community factors that impact the likelihood of receiving a penalty under the Hospital Readmission Reduction Program. This study examined the association between structural, performance, and community factors and the likelihood of receiving a penalty as well as investigated the likelihood of hospitals serving vulnerable populations of receiving a penalty.

**Methods:** Centers for Medicare and Medicaid Services and United States Census Bureau data were used in this analysis. Ordered logistic regressions in a cross-sectional analysis were employed to estimate the probability of receiving a high or low penalty in the fiscal year 2013 through 2019.

**Results:** On average, medium-sized, major teaching, and safety-net hospitals had the highest proportion of hospitals with a high penalty. After controlling for performance and community factors, structural factor variables such as safety-net status, rural status, and teaching status either were no longer significant or the likelihood magnitude changed. However, after controlling for performance and community factors, the statistical significance of hospital size variables and geographic location persisted across the years. Length of stay and occupancy rate variables were also statistically significant across the 7 years under review.

**Conclusion:** Taken together, structural, performance, and community factors are important in explaining variation in the likelihood of receiving a penalty. There is no evidence that safety-net, rural, and public hospitals are more likely to receive a penalty. The results also suggest that there is room for providers to reduce avoidable readmissions and policymakers to mitigate unintended consequences.

## Introduction

The Hospital Readmission Reduction Program (HRRP) is a Centers of Medicare and Medicaid (CMS) incentive program authorized under the Affordable Care Act. The objective of the program is to reduce avoidable readmission among Medicare fee-for-service beneficiaries by penalizing hospitals that have higher than expected readmissions. HRRP was created as a response to concerns regarding adverse patient outcomes, high cost associated with readmission, and lack of incentives for hospitals to tackle the burden of readmissions.^1,2^ Previous studies and advisories to Congress recognized excess readmissions as a signal for suboptimal inpatient care, lack of care coordination, and lack of appropriate care transitions.^1,3^ The program initially targeted three conditions—acute myocardial infarction, heart failure, and pneumonia.

Previous research has shown mixed findings on the effectiveness of the program. Some studies have illustrated that HRRP implementation led to a decline in readmission rates of both targeted and untargeted conditions, suggesting a spillover effect.^4–9^ Others found that HRRP may disproportionately penalize safety-net, medium, and large-sized hospitals.^10–13^ Yet other studies questioned the magnitude of the decline, identified unintended consequences of the program, and recommended changes to the program, including controlling for social risk factors.^12,14–16^

Based on previous work, the factors that are associated with readmissions and therefore the likelihood of being penalized can be broken down into three categories: structural, performance, and community.^17–19^ Structural factors include hospital size, teaching status, and ownership status. Performance factors are metrics that measure a process or outcome, such as operating margin, occupancy rate, patient experience, and 30-day mortality. Community factors include socioeconomic measures, including geographic location and county-level population characteristics.

Recent efforts to enhance HRRP have focused on considerations to control for social factors. For example, literature indicates the importance of controlling for community factors such as the level of neighborhood disadvantage.^12,13,20,21^ Beginning in fiscal year (FY) 2019, penalties were assessed based on the hospital's performance as compared with peers treating a similar proportion of both Medicare and Medicaid eligible patients (dual eligible).^22^ The stratification by dual enrollment status was one of the changes mandated by the 21st Century Cures Act.^22^ Although there are considerations to control for social risk factors, less attention has focused on the impact of hospital performance factors on readmission and the likelihood of receiving a penalty. More importantly, hospital administrators may have some control over performance factors, but not over community factors.^23^ Hospital administrators' control is limited to hospital performance factors. However, little is known about the association between structural, performance, and community factors, and the likelihood of a hospital receiving a penalty. This article sought to investigate the role of structural, performance, and community factors in predicting a hospital's likelihood of receiving a penalty across the 7 years of the HRRP.

## Methods

### Data sources and variables

Publicly available data from CMS files and the United States Census Bureau were used in this analysis. Using the HRRP supplemental data, we divided hospitals into three groups—high penalty recipients (top 50% of those receiving a penalty), low penalty recipients (bottom 50% of those receiving a penalty), and those not receiving any penalty. Using the Impact File data, the study defines a safety-net hospital as the top quartile of Disproportionate Share Hospital Patient Percentage, a measure used to adjust payments for hospitals that serve a disproportionate number of low-income patients. Hospital characteristics, such as ownership, rural/urban indicator, geographic region, technology and services offered (to calculate Saidin Index, measuring high technology/rare services), teaching status, and hospital size, were sourced from the provider of service file.

Hospital operating margin, length of stay, and high occupancy rate (top quartile) variables were sourced from the cost reports. Three patient experience measures (communication about medicines, discharge information, and willingness to recommend the hospital) were extracted from the Hospital Consumer Assessment of Health care Providers and System survey. We also used pneumonia, heart failure, and acute myocardial infarction 30-day mortality rates in the analysis, which were sourced from Hospital Compare database. The study calculated the Herfindahl-Hirschman Index, a measure of hospital market concentration, using the hospital service area file. Population variables, such as those used to calculate Area Deprivation Index (ADI) (which measures the social and economic disadvantage of a neighborhood) and most segregated (first quartile of the multigroup entropy index, which is a measure of residential segregation), were sourced from the American Community Survey (for years 2007–2011 to 2013–2017).^24^ ADI was constructed by using the Singh method at the county level.^25–27^ Based on previous work in this area,^26^ the most disadvantaged areas were defined as the top 15% of the index. Further, the most disadvantaged group was divided into three equal groups representing most disadvantaged, second most disadvantaged, and third most disadvantaged.

The data were aligned to correspond to the performance years under the HRRP program. For example, for the FY 2019, the data are from 2017, which covers three performance years: 2014, 2015, and 2016.

### Statistical analysis

To determine the explanatory variables used in this analysis, we used factor analysis in an iterative process. From an initial list of 53 explanatory variables based on theory and previous work, 32 variables were included in the analysis.^10, 2,13,19,28^ After every iteration, factors with a minimum eigenvalue of 1 and a factor loading above 0.3 were retained. Next, we tested for collinearity by using measures such as variance inflation factor (VIF), eigenvalue, and condition number. Using a threshold of VIF ≤10 and condition number <15, there was no evidence for collinearity concerns (VIF=1.96 and condition number=6.44). We tested for the proportional odds assumption, also known as parallel line assumption. All models failed the proportional odds assumption, primarily due to hospital structural factors. Including higher order variables did not mitigate this issue. Therefore, analyses were conducted with proportional odds models, which relaxes proportionality constraints for only those variables that violate the parallel lines assumptions.^29,30^

After conducting a sensitivity analysis, we determined that there was a potential loss of information because of missing values in several of the variables. We determined that the missing was not Missing Completely at Random and held that the data was Missing at Random. We used Multiple Imputation by Chained Equations to impute operating margin, length of stay, medication communication, recovery information, hospital recommendation, pneumonia 30-day mortality, heart failure 30-day mortality, and multigroup entropy index. The missing values, on average, accounted for 4.8% of the total observations. Safety-net, public, for-profit, rural, hospital size, and teaching status variables were used as auxiliary variables. The study also controlled for county-level clustering of the standard errors.

The study uses a two-stage approach to investigate the role structural factors, performance, and community factors on the likelihood of receiving a penalty. Using generalized ordered logistic regressions (a cross-sectional analysis was chosen because, over the years, CMS had changed how it measured and calculated penalties), we first estimated the association by including only structural factors to determine which factors were significant. We then estimated a model with performance and community factors as control variables. Average marginal effects are reported.

All analyses were conducted by using Stata 15.1. The Institutional Review Board determined that the study did not meet the federal definitions of research involving human participants (UMCIRB 19-002449).

## Results

Table 1 reports the level of a penalty during each FY based on structural factors. Most hospitals received either a high or low penalty throughout the study period. On average, a high proportion of medium-sized, major teaching, and safety-net received a high penalty. Compared with large and medium hospitals, small hospitals had the lowest percentage of hospitals receiving a high penalty, as compared with medium and large hospitals.

**Table 1.** Frequency and Proportion of Hospitals Receiving High, Low, or No Penalty by Structural Factors

		Level of penalty (% in parentheses)			
Year	Hospital characteristic	No penalty	Low penalty	High penalty	Total
2013	Safety-net hospital	225 (26)	307 (35)	343 (39)	875
	Public hospital	255 (40)	207 (32)	175 (27)	637
	For-profit	309 (39)	242 (31)	241 (30)	792
	Not-for-profit	721 (35)	675 (33)	674 (33)	2070
	Rural hospital	364 (38)	255 (26)	346 (36)	965
	Small	612 (52)	277 (24)	289 (25)	1178
	Medium	527 (30)	649 (37)	598 (34)	1774
	Large	147 (27)	198 (36)	203 (37)	548
	Major teaching	110 (25)	151 (35)	173 (40)	434
	Minor teaching	176 (35)	182 (36)	151 (30)	509
2014	Safety-net hospital	222 (26)	321 (37)	327 (38)	870
	Public hospital	236 (37)	208 (33)	186 (30)	630
	For-profit	303 (39)	235 (30)	246 (31)	784
	Not-for-profit	717 (35)	697 (34)	653 (32)	2067
	Rural hospital	339 (35)	267 (28)	353 (37)	959
	Small	573 (49)	296 (25)	303 (26)	1172
	Medium	544 (31)	625 (35)	597 (34)	1766
	Large	141 (26)	219 (40)	185 (34)	545
	Major teaching	108 (25)	165 (38)	158 (37)	431
	Minor teaching	189 (37)	164 (32)	153 (30)	506
2015	Safety-net hospital	151 (17)	375 (43)	343 (39)	869
	Public hospital	149 (24)	280 (45)	189 (31)	618
	For-profit	220 (28)	252 (32)	320 (40)	792
	Not-for-profit	465 (23)	798 (39)	799 (39)	2062
	Rural hospital	205 (22)	362 (38)	383 (40)	950
	Small	420 (36)	386 (33)	360 (31)	1166
	Medium	333 (19)	672 (38)	755 (43)	1760
	Large	85 (15)	272 (49)	193 (35)	550
	Major teaching	78 (18)	191 (43)	173 (39)	442
	Minor teaching	121 (23)	207 (40)	195 (37)	523
2016	Safety-net hospital	134 (16)	368 (44)	343 (41)	845
	Public hospital	155 (25)	261 (43)	198 (32)	614
	For-profit	236 (29)	261 (32)	321 (39)	818
	Not-for-profit	404 (20)	816 (40)	806 (40)	2026
	Rural hospital	242 (25)	350 (36)	386 (39)	978
	Small	460 (38)	383 (32)	360 (30)	1203
	Medium	269 (16)	699 (40)	759 (44)	1727
	Large	70 (13)	258 (48)	206 (39)	534
	Major teaching	58 (13)	199 (45)	182 (41)	439
	Minor teaching	125 (23)	200 (37)	211 (39)	536
2017	Safety-net hospital	130 (15)	371 (44)	339 (40)	840
	Public hospital	166 (28)	247 (41)	184 (31)	597
	For-profit	265 (32)	229 (28)	334 (40)	828
	Not-for-profit	416 (21)	828 (41)	774 (38)	2018
	Rural hospital	266 (28)	341 (35)	355 (37)	962
	Small	516 (43)	352 (29)	326 (27)	1194
	Medium	281 (16)	677 (39)	763 (44)	1721
	Large	53 (10)	275 (52)	205 (38)	533
	Major teaching	62 (14)	207 (48)	166 (38)	435
	Minor teaching	124 (23)	224 (41)	193 (36)	541
2018	Safety-net hospital	122 (15)	377 (45)	331 (40)	830
	Public hospital	154 (27)	243 (43)	172 (30)	569
	For-profit	239 (29)	228 (28)	348 (43)	815
	Not-for-profit	382 (19)	844 (42)	797 (39)	2023
	Rural hospital	236 (27)	333 (38)	311 (35)	880
	Small	490 (42)	364 (31)	317 (27)	1171
	Medium	239 (14)	690 (40)	781 (46)	1710
	Large	51 (10)	263 (49)	219 (41)	533
	Major teaching	51 (12)	201 (46)	181 (42)	433
	Minor teaching	113 (20)	228 (41)	215 (39)	556
2019	Safety-net hospital	122 (15)	387 (49)	287 (36)	796
	Public hospital	107 (21)	232 (46)	167 (33)	506
	For-profit	165 (22)	229 (31)	342 (46)	736
	Not-for-profit	298 (15)	842 (44)	786 (41)	1926
	Rural hospital	171 (22)	334 (42)	284 (36)	789
	Small	339 (34)	352 (35)	312 (31)	1003
	Medium	186 (11)	686 (42)	765 (47)	1637
	Large	49 (9)	266 (50)	218 (41)	533
	Major teaching	45 (11)	209 (50)	166 (40)	420
	Minor teaching	86 (16)	231 (43)	217 (41)	534

The average marginal effect of structural factors on probability of receiving a high/low penalty is reported in Table 2. In 2013, a safety-net hospital was associated with a 13.9% (*p*<0.001) point increase in the predicted probability of receiving a high penalty as compared with a non-safety-net hospital. This finding decreased in magnitude and significance in later years, except in 2019, when a safety-net hospital was associated with a 4.6% (*p*<0.001) point decrease in the predicted probability of receiving a high penalty. In 2019, the predicted probability of receiving a low penalty for a safety-net hospital increased by 7.8% (*p*<0.001) points compared with a non-safety-net hospital, an increase in magnitude from the previous 2 years. Medium and large hospitals are consistently more likely to receive a high penalty throughout the study period, compared with small hospitals. In addition to safety-net, rural (vs. urban), medium and large (vs. small) hospitals were found to be more likely to receive a high penalty, but they had a lower probability of receiving a low penalty in some years. Hospital geographic region was also statistically significant in predicting hospitals' likelihood of receiving either high or low penalty, controlling for other structural factors.

**Table 2.** Average Marginal Effects of Structural Factors on Probability of Receiving a High or Low Penalty (Fiscal Year 2013–2019)

	High penalty	FY 2013	FY 2014	FY 2015	FY 2016	FY 2017	FY 2018	FY 2019
	Non-safety-net hospital	Ref	Ref	Ref	Ref	Ref	Ref	Ref
Structural factors	Safety-net hospital	0.139^***^ (0.020)	0.114^***^ (0.023)	0.048^*^ (0.022)	0.047 (0.024)	0.051^*^ (0.025)	0.028 (0.023)	−0.046^*^ (0.021)
	Not-for-profit							
	Public	−0.022 (0.019)	−0.007 (0.019)	−0.047^*^ (0.024)	−0.033 (0.021)	−0.044^*^ (0.020)	−0.048^*^ (0.021)	−0.024 (0.022)
	For-profit	0.013 (0.020)	0.016 (0.020)	0.056^*^ (0.023)	0.035 (0.022)	0.040 (0.023)	0.060^**^ (0.022)	0.076^**^ (0.024)
	Urban hospital	Ref	Ref	Ref	Ref	Ref	Ref	Ref
	Rural hospital	0.114^***^ (0.022)	0.095^***^ (0.021)	0.069^**^ (0.022)	0.065^**^ (0.020)	0.055^**^ (0.021)	0.012 (0.022)	−0.027 (0.024)
	Small (<100 beds)	Ref	Ref	Ref	Ref	Ref	Ref	Ref
	Medium (100–399 beds)	0.118^***^ (0.020)	0.107^***^ (0.019)	0.134^***^ (0.020)	0.153^***^ (0.019)	0.188^***^ (0.019)	0.185^***^ (0.019)	0.157^***^ (0.021)
	Large (400+ beds)	0.152^***^ (0.033)	0.114^***^ (0.032)	0.077^*^ (0.031)	0.110^***^ (0.032)	0.163^***^ (0.030)	0.160^***^ (0.030)	0.128^***^ (0.031)
	Non-teaching	Ref	Ref	Ref	Ref	Ref	Ref	Ref
	Major teaching	−0.003 (0.024)	−0.009 (0.025)	−0.038 (0.028)	−0.012 (0.028)	−0.060^*^ (0.025)	−0.034 (0.023)	−0.062^*^ (0.025)
	Minor teaching	−0.034 (0.018)	−0.043^*^ (0.018)	−0.039 (0.022)	−0.016 (0.024)	−0.063^**^ (0.019)	−0.047^*^ (0.020)	−0.047^*^ (0.021)
	West	Ref	Ref	Ref	Ref	Ref	Ref	Ref
	Northeast	0.407^***^ (0.034)	0.364^***^ (0.032)	0.341^***^ (0.028)	0.293^***^ (0.028)	0.238^***^ (0.030)	0.287^***^ (0.029)	0.283^***^ (0.030)
	Midwest	0.195^***^ (0.045)	0.168^***^ (0.042)	0.179^***^ (0.031)	0.169^***^ (0.030)	0.106^***^ (0.030)	0.116^***^ (0.028)	0.113^***^ (0.030)
	South	0.201^***^ (0.031)	0.201^***^ (0.029)	0.172^***^ (0.025)	0.177^***^ (0.023)	0.153^***^ (0.024)	0.194^***^ (0.022)	0.197^***^ (0.022)
	**Low penalty**	**FY 2013**	**FY 2014**	**FY 2015**	**FY 2016**	**FY 2017**	**FY 2018**	**FY 2019**
	**Non-safety hospital**	**Ref**	**Ref**	**Ref**	**Ref**	**Ref**	**Ref**	**Ref**
Structural factors	Safety-net hospital	−0.004 (0.006)	−0.002 (0.005)	0.033 (0.022)	0.026 (0.023)	0.045^*^ (0.022)	0.053^**^ (0.020)	0.078^***^ (0.022)
	Not-for-profit	Ref	Ref	Ref	Ref	Ref	Ref	Ref
	Public	−0.002 (0.002)	−0.000 (0.001)	0.055^*^ (0.023)	0.009 (0.005)	0.011^*^ (0.005)	0.014^*^ (0.006)	0.009 (0.009)
	For-profit	0.001 (0.001)	0.001 (0.001)	−0.064^**^ (0.020)	−0.082^***^ (0.020)	−0.101^***^ (0.018)	−0.111^***^ (0.019)	−0.100^***^ (0.022)
	Rural hospital	−0.046^*^ (0.018)	−0.001 (0.003)	0.019 (0.020)	−0.021^**^ (0.007)	−0.016^*^ (0.007)	0.035 (0.020)	0.056^**^ (0.021)
	Small (<100 beds)	Ref	Ref	Ref	Ref	Ref	Ref	Ref
	Medium (100–399 beds)	0.103^***^ (0.018)	0.090^***^ (0.018)	0.048^*^ (0.019)	0.059^***^ (0.018)	0.055^**^ (0.019)	0.068^***^ (0.019)	0.054^**^ (0.020)
	Large (400+ beds)	0.060^*^ (0.027)	0.107^***^ (0.027)	0.104^***^ (0.028)	0.076^**^ (0.028)	0.073^**^ (0.028)	0.058^*^ (0.027)	0.045 (0.028)
	Non-teaching	Ref	Ref	Ref	Ref	Ref	Ref	Ref
	Major teaching	−0.000 (0.002)	−0.001 (0.002)	0.008 (0.005)	0.003 (0.008)	0.014^**^ (0.005)	0.010 (0.006)	0.023^**^ (0.008)
	Minor teaching	−0.003 (0.003)	−0.004 (0.004)	0.008^*^ (0.004)	−0.047^*^ (0.023)	0.014^***^ (0.004)	0.014^**^ (0.005)	0.018^*^ (0.007)
	**West**	**Ref**	**Ref**	**Ref**	**Ref**	**Ref**	**Ref**	**Ref**
	Northeast	−0.127^***^ (0.025)	−0.119^***^ (0.027)	−0.149^***^ (0.016)	−0.131^***^ (0.017)	−0.096^***^ (0.016)	−0.132^***^ (0.018)	−0.154^***^ (0.020)
	Midwest	−0.071^*^ (0.028)	−0.064^*^ (0.028)	−0.058^***^ (0.013)	−0.092^***^ (0.021)	−0.034^**^ (0.011)	−0.042^***^ (0.012)	−0.022 (0.024)
	South	−0.043^*^ (0.022)	−0.058^*^ (0.025)	−0.043^***^ (0.009)	−0.053^***^ (0.009)	−0.044^***^ (0.008)	−0.064^***^ (0.009)	−0.084^***^ (0.011)
	Observations	3499	3481	3472	3459	3446	3411	3173

^*^p<0.05, ^**^p<0.01, ^***^p<0.001.

Standard errors in parentheses.

FY, fiscal year.

Table 3 displays the average marginal effects of structural, performance, and community factors on the probability of receiving high penalty. In 2013 and 2016, a safety-net hospital status was associated with a 3.5% and 4.1% (*p*<0.05) point increase in the predicted probability of receiving a high penalty, respectively, compared with a non-safety-net hospital. The safety-net hospital indicator was not statistically significant over the other years. Medium and large hospitals were positively and statistically significantly associated with the likelihood of receiving a high penalty compared with small hospitals, after holding structural, performance, and community factors constant.

**Table 3.** Average Marginal Effects of Receiving a High Penalty (Fiscal Year 2013–2019)

		FY 2013	FY 2014	FY 2015	FY 2016	FY 2017	FY 2018	FY 2019
Structural factors	Safety-net hospital	0.035^*^ (0.017)	0.004 (0.019)	0.023 (0.019)	0.041^*^ (0.020)	0.016 (0.023)	−0.017 (0.021)	−0.020 (0.021)
	Not for profit	Ref	Ref	Ref	Ref	Ref	Ref	Ref
	Public	−0.024 (0.017)	−0.018 (0.017)	−0.054^*^ (0.022)	−0.028 (0.020)	−0.045^*^ (0.020)	−0.039^*^ (0.020)	−0.015 (0.022)
	For-profit	0.000 (0.020)	0.003 (0.018)	0.024 (0.020)	−0.002 (0.021)	0.000 (0.021)	0.007 (0.020)	0.021 (0.022)
	Urban hospital	Ref	Ref	Ref	Ref	Ref	Ref	Ref
	Rural hospital	0.041 (0.024)	−0.000 (0.021)	0.002 (0.023)	−0.015 (0.023)	−0.022 (0.023)	−0.034 (0.024)	−0.024 (0.026)
	Small (<100 beds)	Ref	Ref	Ref	Ref	Ref	Ref	Ref
	Medium (100–399 beds)	0.100^***^ (0.017)	0.092^***^ (0.017)	0.125^***^ (0.019)	0.167^***^ (0.019)	0.177^***^ (0.019)	0.204^***^ (0.019)	0.194^***^ (0.022)
	Large (400+ beds)	0.136^***^ (0.029)	0.148^***^ (0.027)	0.149^***^ (0.030)	0.172^***^ (0.031)	0.182^***^ (0.030)	0.207^***^ (0.031)	0.185^***^ (0.034)
	Non-teaching	Ref	Ref	Ref	Ref	Ref	Ref	Ref
	Major teaching	0.001 (0.021)	−0.005 (0.023)	−0.028 (0.025)	−0.011 (0.025)	−0.064^**^ (0.024)	−0.014 (0.020)	−0.044 (0.023)
	Minor teaching	−0.033^*^ (0.016)	−0.040^*^ (0.016)	−0.035 (0.020)	−0.017 (0.023)	−0.050^**^ (0.018)	−0.025 (0.018)	−0.032 (0.020)
Performance factors	Operating margin	−0.000^***^ (0.000)	−0.000^*^ (0.000)	−0.001^***^ (0.000)	−0.001^***^ (0.000)	−0.001^***^ (0.000)	−0.001^***^ (0.000)	−0.001^***^ (0.000)
	Length of stay	−0.027^***^ (0.005)	−0.022^***^ (0.004)	−0.063^***^ (0.015)	−0.057^***^ (0.013)	−0.040^***^ (0.007)	−0.043^***^ (0.006)	−0.078^***^ (0.011)
	Average low occupancy rate (<66%)	Ref	Ref	Ref	Ref	Ref	Ref	Ref
	Average high occupancy (66–99%)	0.063^***^ (0.017)	0.064^***^ (0.017)	0.048^*^ (0.020)	0.073^***^ (0.018)	0.039 (0.020)	0.026 (0.020)	0.098^***^ (0.021)
	Medication communication	−0.005^*^ (0.002)	−0.006^**^ (0.002)	−0.005^*^ (0.003)	0.001 (0.003)	0.004 (0.003)	0.007 (0.004)	0.002 (0.004)
	Recovery information	−0.013^***^ (0.002)	−0.010^***^ (0.002)	−0.001 (0.003)	−0.001 (0.003)	−0.009^*^ (0.004)	−0.021^***^ (0.003)	−0.013^**^ (0.004)
	Recommend hospital	−0.004^***^ (0.001)	−0.006^***^ (0.001)	−0.006^***^ (0.001)	−0.008^***^ (0.002)	−0.003 (0.002)	−0.003 (0.003)	−0.001 (0.003)
	Pneumonia 30-day mortality	0.000 (0.004)	0.000 (0.004)	−0.000 (0.004)	−0.006 (0.005)	0.030^***^ (0.004)	0.029^***^ (0.004)	0.028^***^ (0.004)
	Heart failure 30-day mortality	−0.018^***^ (0.005)	−0.015^***^ (0.004)	−0.007 (0.006)	−0.006 (0.006)	−0.019^**^ (0.006)	−0.025^***^ (0.006)	−0.025^***^ (0.006)
	HHI	0.028 (0.024)	0.059^*^ (0.026)	0.038 (0.030)	0.042 (0.029)	0.023 (0.025)	0.037 (0.028)	0.013 (0.029)
Community factors	Saidin Index	0.000 (0.001)	−0.001 (0.001)	0.000 (0.001)	0.001 (0.001)	0.001 (0.001)	0.001 (0.001)	0.001 (0.001)
	Low share of Medicare	Ref	Ref	Ref	Ref	Ref	Ref	Ref
	High share of Medicare	0.057^**^ (0.018)	0.061^***^ (0.018)	0.040^*^ (0.019)	0.082^***^ (0.022)	0.080^***^ (0.019)	0.066^***^ (0.020)	0.067^**^ (0.023)
	Wage Index for FY	0.045 (0.058)	0.078 (0.047)	0.153^**^ (0.059)	0.008 (0.050)	0.024 (0.053)	−0.040 (0.050)	−0.074 (0.050)
	Low comorbidities rate	Ref	Ref	Ref	Ref	Ref	Ref	Ref
	High comorbidities rate	0.083^**^ (0.031)	0.074^*^ (0.034)	0.063^*^ (0.029)	0.027 (0.023)	0.070^**^ (0.023)	0.046 (0.024)	0.062^*^ (0.024)
	Low ED utilization rate	Ref	Ref	Ref	Ref	Ref	Ref	Ref
	High ED utilization rate	0.101^***^ (0.023)	0.086^***^ (0.022)	0.054^*^ (0.024)	0.039 (0.022)	0.075^***^ (0.020)	0.047^*^ (0.020)	0.017 (0.022)
	Low minority percent	Ref	Ref	Ref	Ref	Ref	Ref	Ref
	High minority percent	−0.044 (0.030)	−0.046 (0.029)	−0.068^*^ (0.029)	−0.070^**^ (0.024)	−0.077^**^ (0.025)	−0.071^**^ (0.025)	−0.076^**^ (0.028)
	Multigroup entropy index	0.002 (0.002)	0.001 (0.001)	0.001 (0.002)	0.001 (0.002)	0.002 (0.002)	0.001 (0.002)	0.001 (0.002)
	Average HCC Score	−0.039 (0.167)	−0.062 (0.172)	−0.062 (0.180)	0.074 (0.163)	0.232 (0.157)	0.154 (0.138)	0.100 (0.141)
	ADI (bottom 85%)	Ref	Ref	Ref	Ref	Ref	Ref	Ref
	ADI-Most disadvantaged	0.142^***^ (0.036)	0.181^***^ (0.038)	0.075 (0.039)	−0.005 (0.036)	−0.019 (0.038)	−0.005 (0.036)	−0.047 (0.040)
	ADI-Second most disadvantaged	0.086^**^ (0.030)	0.110^***^ (0.032)	0.112^***^ (0.034)	0.054 (0.037)	−0.012 (0.031)	0.018 (0.035)	0.010 (0.036)
	ADI-Third most disadvantaged	0.042 (0.028)	0.025 (0.027)	0.009 (0.031)	0.030 (0.030)	0.039 (0.034)	0.012 (0.031)	0.013 (0.035)
	Medicaid Adults Eligibility (100% FPL+)	−0.016 (0.026)	−0.011 (0.027)	−0.022 (0.030)	0.062^***^ (0.018)	0.047^*^ (0.021)	0.038 (0.024)	0.016 (0.024)
	West	Ref	Ref	Ref	Ref	Ref	Ref	Ref
	Northeast	0.274^***^ (0.033)	0.219^***^ (0.034)	0.269^***^ (0.033)	0.210^***^ (0.035)	0.140^***^ (0.034)	0.196^***^ (0.033)	0.200^***^ (0.035)
	Midwest	0.127^***^ (0.033)	0.090^**^ (0.030)	0.149^***^ (0.034)	0.105^**^ (0.034)	0.044 (0.031)	0.032 (0.033)	0.031 (0.034)
	South	0.127^***^ (0.032)	0.107^**^ (0.033)	0.156^***^ (0.036)	0.170^***^ (0.032)	0.091^**^ (0.033)	0.106^**^ (0.033)	0.110^***^ (0.033)
	Observations	3499	3481	3472	3459	3446	3411	3173

^*^ *p*<0.05, ^**^p<0.01, ^***^p<0.001.

Standard errors in parentheses.

HHI, Herfindahl-Hirschman Index; ED, emergency department; HCC, Hierarchical Condition Category; ADI, Area Deprivation Index; FPL, Federal poverty level.

Aside from structural factors, performance factors such as length of stay, average high occupancy, high proportion of Medicare patients, patient experience measures (medication, patient recovery, and patient hospital recommendation), and operating margin were statistically significant in predicting the probability of receiving a high penalty. For example, in 2013, a 1-day increase in the length of stay was associated with a 2.7% (*p*<0.001) point decrease in the predicted probability of receiving a high penalty. By 2019, a 1-day increase in the length of stay was associated with a 7.8% (*p*<0.001) point decrease in the probability of receiving a high penalty. Average high occupancy rate (≥66%) had a positive and statistically significant association with receiving a high penalty for much of the study period. The increase in probability ranged from 2.6% points to 9.8% (*p*<0.001) points. Patient experience measures were associated with decreases in the predicated probabilities of receiving a high penalty in most of the years, suggesting that patients who had a positive experience were less likely to be readmitted within 30 days.

Several community factors had a statistically significant association with the probability of receiving a high penalty, including hospitals located in counties with a high emergency department (ED) utilization rate (775–1273 ED visits per 100,000 residents), counties with high comorbidities rate (16.7–32.45% of residents with six or more comorbidities), and county-level disadvantage. After controlling for performance and community factors, geographic region continued to have a strong association with earning a high penalty, suggesting geographic location as a predictor of variation in the likelihood of receiving a high penalty. Also, in the past 5 years, hospitals located in counties with a high proportion of minorities (≥50%) were associated with a decreased predicted probability of receiving a high penalty (Table 3).

Finally, the results in Table 4 indicate that medium- and large-sized hospitals are less likely to receive a low penalty as compared with small-sized hospitals. The findings were consistent in that the factors that statistically contributed to a hospital being likely to receive a high penalty were also statistically significant (in the opposite direction) for contributing to the likelihood of receiving a low penalty. The results suggest that hospitals that are more likely to receive a high penalty are also less likely to receive a low penalty.

**Table 4.** Average Marginal Effects of Receiving a Low Penalty (Fiscal Year 2013–2019)

		FY 2013	FY 2014	FY 2015	FY 2016	FY 2017	FY 2018	FY 2019
	Non-safety-net hospital	Ref	Ref	Ref	Ref	Ref	Ref	Ref
Structural factors	Safety-net hospital	0.002 (0.001)	0.054^**^ (0.019)	−0.006 (0.005)	−0.012 (0.006)	0.067^**^ (0.021)	0.088^***^ (0.020)	0.007 (0.008)
	Not for profit	Ref	Ref	Ref	Ref	Ref	Ref	Ref
	Public	−0.002 (0.002)	−0.001 (0.002)	0.068^**^ (0.022)	0.007 (0.005)	0.012^*^ (0.005)	0.011^*^ (0.005)	0.006 (0.008)
	For-profit	0.013 (0.020)	0.000 (0.001)	−0.006 (0.006)	0.001 (0.006)	−0.000 (0.006)	−0.002 (0.006)	−0.008 (0.009)
	Urban hospital	Ref	Ref	Ref	Ref	Ref	Ref	Ref
	Rural hospital	−0.071^***^ (0.018)	−0.000 (0.001)	−0.001 (0.006)	0.004 (0.006)	0.006 (0.006)	0.010 (0.006)	0.009 (0.009)
	Small (<100 beds)	Ref	Ref	Ref	Ref	Ref	Ref	Ref
	Medium (100–399 beds)	0.009^*^ (0.004)	0.007 (0.004)	−0.029^***^ (0.005)	−0.042^***^ (0.006)	−0.049^***^ (0.006)	−0.057^***^ (0.007)	−0.071^***^ (0.008)
	Large (400+ beds)	−0.005 (0.006)	−0.009 (0.006)	−0.049^***^ (0.012)	−0.063^***^ (0.014)	−0.070^***^ (0.014)	−0.084^***^ (0.015)	−0.087^***^ (0.018)
	Non-teaching	Ref	Ref	Ref	Ref	Ref	Ref	Ref
	Major teaching	0.000 (0.001)	−0.000 (0.002)	0.006 (0.005)	0.003 (0.006)	0.082^**^ (0.025)	0.004 (0.005)	0.015^*^ (0.007)
	Minor teaching	−0.003 (0.003)	−0.004 (0.003)	0.007^*^ (0.004)	−0.021 (0.023)	0.013^**^ (0.004)	0.007 (0.005)	0.012 (0.007)
Performance factors	Operating margin	−0.000^**^ (0.000)	−0.000^*^ (0.000)	0.000 (0.000)	0.000^*^ (0.000)	0.000^***^ (0.000)	0.000^***^ (0.000)	0.000^***^ (0.000)
	Length of stay	−0.002^*^ (0.001)	−0.001 (0.001)	0.040^**^ (0.014)	0.031^**^ (0.012)	0.012^***^ (0.002)	0.013^***^ (0.002)	0.059^***^ (0.011)
	Average low occupancy rate (<66%)	Ref	Ref	Ref	Ref	Ref	Ref	Ref
	Average high occupancy (66–99%)	0.002 (0.002)	0.001 (0.002)	−0.013^*^ (0.006)	−0.022^***^ (0.007)	0.082^***^ (0.021)	0.084^***^ (0.020)	−0.042^***^ (0.010)
	Medication communication	−0.000 (0.000)	−0.000 (0.000)	0.001 (0.001)	−0.000 (0.001)	−0.001 (0.001)	−0.001 (0.002)	−0.001 (0.001)
	Recovery information	−0.001 (0.001)	−0.001 (0.000)	−0.005 (0.003)	−0.005 (0.003)	0.003^*^ (0.001)	0.006^***^ (0.001)	0.001 (0.003)
	Recommend hospital	−0.000 (0.000)	−0.000 (0.000)	0.002^***^ (0.000)	0.002^***^ (0.000)	0.001 (0.001)	0.001 (0.001)	0.000 (0.001)
	Pneumonia 30-day mortality	0.000 (0.000)	0.000 (0.000)	0.000 (0.001)	0.002 (0.001)	0.013^***^ (0.004)	−0.009^***^ (0.001)	−0.011^***^ (0.002)
	Heart failure 30-day mortality	−0.001 (0.001)	−0.001 (0.001)	0.002 (0.001)	0.002 (0.002)	0.006^**^ (0.002)	0.007^***^ (0.002)	0.005 (0.006)
	HHI	0.003 (0.002)	−0.072^***^ (0.020)	−0.040 (0.023)	−0.024 (0.023)	−0.006 (0.007)	−0.000 (0.023)	−0.004 (0.010)
Community factors	Saidin Index	0.000 (0.000)	−0.000 (0.000)	−0.000 (0.000)	−0.000 (0.000)	−0.000 (0.000)	−0.000 (0.000)	−0.001 (0.001)
	Low share of Medicare	Ref	Ref	Ref	Ref	Ref	Ref	Ref
	High share of Medicare	0.004 (0.002)	0.003 (0.002)	−0.010^*^ (0.005)	−0.062^**^ (0.021)	−0.023^***^ (0.006)	−0.020^**^ (0.006)	−0.086^***^ (0.021)
	Wage Index for FY	0.095^*^ (0.047)	0.004 (0.004)	−0.037^*^ (0.015)	−0.002 (0.013)	−0.007 (0.016)	0.012 (0.014)	0.027 (0.018)
	Low comorbidities rate	Ref	Ref	Ref	Ref	Ref	Ref	Ref
	High comorbidities rate	−0.002 (0.004)	−0.001 (0.003)	−0.018 (0.009)	−0.007 (0.007)	−0.023^**^ (0.009)	−0.015 (0.009)	−0.025^*^ (0.010)
	Low ED utilization rate							
	High ED utilization rate	−0.054^**^ (0.020)	−0.000 (0.003)	−0.015 (0.008)	−0.011 (0.007)	−0.024^**^ (0.007)	−0.015^*^ (0.007)	−0.007 (0.009)
	Low minority percent	Ref	Ref	Ref	Ref	Ref	Ref	Ref
	High minority percent	−0.005 (0.005)	−0.004 (0.005)	0.013^**^ (0.005)	0.016^***^ (0.004)	0.019^***^ (0.006)	0.018^**^ (0.006)	0.026^**^ (0.009)
	Multigroup entropy index	0.000 (0.000)	0.000 (0.000)	−0.000 (0.000)	−0.000 (0.000)	−0.001 (0.000)	−0.000 (0.000)	−0.000 (0.001)
	Average HCC Score	−0.002 (0.008)	−0.002 (0.006)	0.016 (0.046)	−0.021 (0.045)	−0.086 (0.106)	−0.048 (0.043)	−0.039 (0.056)
	ADI (bottom 85%)	Ref	Ref	Ref	Ref	Ref	Ref	Ref
	ADI-Most disadvantaged	−0.011 (0.008)	−0.139^***^ (0.026)	−0.023 (0.014)	0.001 (0.009)	0.005 (0.010)	0.001 (0.010)	0.016 (0.012)
	ADI-Second most disadvantaged	−0.002 (0.004)	−0.007 (0.006)	−0.037^**^ (0.014)	−0.079^*^ (0.032)	0.004 (0.008)	−0.006 (0.011)	−0.004 (0.014)
	ADI-Third most disadvantaged	0.001 (0.002)	0.001 (0.001)	−0.002 (0.008)	−0.009 (0.009)	−0.013 (0.012)	−0.004 (0.010)	−0.005 (0.014)
	Medicaid Adults Eligibility (100% FPL+)	−0.001 (0.003)	−0.001 (0.002)	0.005 (0.006)	−0.017^**^ (0.005)	−0.015^*^ (0.007)	0.026 (0.023)	−0.006 (0.009)
	West	Ref	Ref	Ref	Ref	Ref	Ref	Ref
	Northeast	−0.042^**^ (0.014)	−0.028^*^ (0.012)	−0.109^***^ (0.018)	−0.084^***^ (0.018)	−0.053^***^ (0.016)	−0.081^***^ (0.018)	−0.099^***^ (0.021)
	Midwest	−0.002 (0.006)	−0.001 (0.004)	−0.046^***^ (0.013)	−0.033^**^ (0.013)	−0.014 (0.011)	0.040 (0.027)	0.042 (0.025)
	South	0.008 (0.004)	0.005 (0.004)	−0.037^***^ (0.010)	−0.046^***^ (0.010)	−0.028^*^ (0.011)	−0.017 (0.024)	−0.043^**^ (0.014)
	Observations	3499	3481	3472	3459	3446	3411	3173

Standard errors in parentheses.

^*^ *p*<0.05, ^**^p<0.01, ^***^p<0.001.

Overall, the evidence suggests that safety-net, rural, teaching, and public hospitals, which typically serve vulnerable populations, do not face a high probability of receiving penalties as compared with non-safety-net, urban, non-teaching, and non-public hospitals.

## Discussion

Overall, the results indicate that controlling for structural factors only may lead to an overestimation of the likelihood of receiving a high penalty for safety-net, rural hospitals, and regional variation, while underestimating the predicted probability for medium and large hospitals. For example, the findings show that safety-net hospitals are likely to receive a high penalty in most of the years when controlling for structural factors only. But after controlling for structural, performance, and community factors, the safety-net hospital has an increase in the predicated probability of receiving a high penalty in only 2 of the 7 years. Further, the magnitude demonstrably changes once the study controls for all factors. In addition, in the first 5 years of the program, rural hospitals are associated with an increased likelihood of receiving a high penalty when only controlling for structural factors. However, when controlling for structural, performance, and community factors, the statistical significance disappears. Therefore, it appears that more inclusive models, with structural, performance, and community factors, are instructive in evaluating such an important policy intervention.

The study finds that medium and large hospitals have an increased probability of receiving a high penalty in all years, a finding that is consistent with a previous study.^10^ The results suggest a connection between structural (hospital size) and performance factors (health care quality) that is associated with high readmissions and, as a result, a high penalty. However, previous evidence is contradictory. Krumholz et al. found that patients admitted to a high performing hospital had significantly lower 30-day readmission rates, suggesting a link between readmissions and quality of care, independent of patient factors.^31^ Tsai et al., who focused on surgical readmissions rates and quality of care, concluded that high-volume hospitals and low mortality had significantly lower readmission rates than low-volume hospitals.^28^ However, in another early study on patients with heart failure, the authors conclude that there was no difference in quality of care and clinical outcomes among hospitals with high versus low risk-adjusted 30-day heart failure readmission rates.^32^ The authors suggest that the findings “raise questions about the validity of the HRRP performance metric in identifying and penalizing low-performance centers.”^32^ A 2017 study concluded that many hospitals experience perpetual penalization and “limited capacity to reduce penalty burden,” suggesting a lack of association between quality performance and penalty.^33^

However, findings in this study show that several performance factors are strongly associated with the likelihood of receiving a penalty. For example, average length of stay, occupancy rate, patient experience measures, and 30-day mortality measures are associated with an often statistically significant increase in the predicted probability of receiving a penalty. The results are consistent with previous studies that have examined specific performance factors and concluded that these factors are associated with high readmission rates.^34–39^ We conclude that variation in the likelihood of receiving a high penalty may be partly explained by these performance factors in addition to structural and community factors. Further, this suggests that hospital administrators exploring pathways to reduce readmission may need to investigate the hospital's performance holistically, including length of stay, occupancy rate, and patient experience measures.

Previous studies have also found a strong association between individual community factors and the risk of readmission and receiving a penalty under HRRP. For example, researchers found that adjusting for social risk impacted the hospital performance and penalties, especially for safety-net hospitals.^12^ Although several factors in this study are associated with an increased risk of receiving a penalty, measures such as area disadvantage are only statistically significant during the first 3 years of the program. Consistent with a previous finding,^40^ we find that a hospital with a high proportion of Medicare patients still faced an increased risk of high penalty.

Also, surprisingly we found that hospitals located in counties with a high minority population, overall, had a decreased likelihood of receiving a high penalty. Multigroup entropy index, a measure of residential segregation, was not significantly associated with a probability of a hospital receiving a high penalty. These findings are contrary to previous reporting of a negative association between racial and ethnic diversity and 30-day readmission for heart failure, acute myocardial infarction, pneumonia, and hip replacements.^41^ Taken together, the findings suggest that hospitals located in counties with a majority of racial and ethnic minority residents perhaps face less risk associated with readmission than minorities in white majority counties.

Overall, the results of this study raise implications and a future direction of inquiry. First, structural, performance, and community factors are important measures in evaluating HRRP. Therefore, those estimating the effect of the HRRP should consider adjusting for structural, performance, and community factors. Researchers and policy makers should not emphasize any one set of factors at the expense of the holistic approach of evaluating potential consequences of the program. Second, from a policy perspective, hospital size and proportion of Medicare fee-for-service patients call for more investigation and possible inclusion in risk adjustment. Lastly, from a hospital administration perspective, average length of stay, patient experience measures, and hospital occupancy rate appear to be good candidates for more scrutiny, and perhaps efforts to improve hospital performance.

## Limitations

The major strength of this study is the study design and methodology, including the choice of structural, performance, and community variables. Also, the longitudinal nature of this study to include all 7 years of the program elicits more information compared with a 1-year cross-sectional analysis. As with any observational study, the present research lacks the ability to report causation. Changes in reporting in Medicare data also represent a significant limitation of this study. The definition and categorization of certain variables such as safety-net and hospital size can be said to be subjective and are decided by the researcher. This can lead to varying research findings and conclusions.

## Conclusion

The structural, performance, and community factors, taken together, are important in explaining variation in hospital penalty levels under the HRRP. Future work by researchers and policy makers need to consider these factors while evaluating the HRRP or working on program redesign. There is little to no evidence suggesting that hospitals serving vulnerable populations have a high probability of receiving a penalty once structural, performance, and community factors are held constant.

## Abbreviations Used

ADI: Area Deprivation Index
CMS: Centers of Medicare and Medicaid
ED: emergency department
FPL: Federal poverty level
FY: fiscal year
HCC: Hierarchical Condition Category
HHI: Herfindahl-Hirschman Index
HRRP: Hospital Readmission Reduction Program
VIF: variance inflation factor
